# Ingestion of maple-based and other carbohydrate sports drinks: effect on sensory perceptions during prolonged exercise

**DOI:** 10.1186/s12970-020-00384-3

**Published:** 2020-12-09

**Authors:** Lorianne Lavoie, Jonathan Tremblay

**Affiliations:** School of Kinesiology and Exercise Science, Faculty of Medicine, University of Montreal, P.O. Box 6128, Downtown Station, Montreal, H3C 3J7 Canada

**Keywords:** Beverage acceptability, Palatability, Maple, Sports drinks, Endurance

## Abstract

**Background:**

Taste and appreciation of sports drinks can affect perceived exertion during exercise. Anecdotal evidence shows that maple products are regularly consumed by recreational and professional athletes but very few studies have reported on their effects during exercise. The purpose of the current study is to report the taste, appreciation and perceived exertion following the ingestion of maple-based sports drinks and other carbohydrate drinks during prolonged exercise.

**Methods:**

Recreationally and competitively active male subjects (*n* = 76, mass = 73.7 ± 10.3 kg, maximum oxygen consumption (VO_2_max) = 4.4 ± 0.5 L/min, maximal aerobic power (MAP) = 309 ± 42 W) ingested one of four carbohydrate solutions (all at 60 g CHO/L): concentrated maple sap (MW), diluted maple syrup (MS), glucose (G), a commercial sports drink (CSD), or a placebo (P; water sweetened with stevia) at every 30 min during 120 min of steady-state exercise (SSE) on a cycle ergometer at 66% MAP. Ratings of perceived exertion (RPE, Borg CR-10) were recorded at each 30 min throughout SSE. A questionnaire was administered to assess sensory characteristics (sweetness, acidity, refreshing, and overall taste on a visual analogue scale, converted to decimals from 0 to 1) and appreciation (sweet, acid and overall on a 9-point hedonic scale) 30 min before (immediately after the first ingestion) and immediately after SSE.

**Results:**

Sweetness was perceived to be higher for MW than G and P (pre: 0.60 ± 0.19, 0.51 ± 0.17 and 0.50 ± 0.17 and post: 0.69 ± 0.19, 0.34 ± 0.18 and 0.48 ± 0.22; *p* < 0.05, respectively) and MS was rated higher than MW for the appreciation of the sweet taste (pre: 6.5 ± 1.5 vs. 4.6 ± 1.8 and post: 6.8 ± 1.8 and 4.1 ± 1.8; *p* < 0.05, respectively). Furthermore, subjects that had ingested MW, reported a significantly lower RPE than those with P at 120 min (14.1 ± 2.2 vs. 16.0 ± 2.0, respectively).

**Conclusions:**

A sports drink containing maple syrup is well appreciated during prolonged exercise and appears to be a viable alternatives to more common sources of carbohydrates.

**Trial registration:**

NCT02880124. Registered on 26 August 2016.

## Introduction

Fluid and carbohydrate intake during endurance events are known to play a major role in maintaining an adequate energy supply for prolonged exercise performance [9]. But the main recommendations and guidelines with regard to the timing and the amount of carbohydrate intake in order to maximize performance [22], as well as the optimal beverage composition for adequate fluid balance [4] don’t consider human-related factors such as changes in sensory perception induced by exercise nor the beverage’s appreciation.

To our knowledge, only a few studies have investigated palatability in an exercise context. Beverage palatability is defined by Passe et al. as “[...] the hedonic evaluation of the sensory properties of the beverage, such as taste, smell, and texture” [20]. Higher palatability can lead to increased voluntary fluid intake [19, 20], and pleasantness was reported to change over the exercise period itself [1, 14]. These factors could be related to what was previously described by Cabanac as the “physiological usefulness” of ingesting a beverage [7].

When assessing the palatability of beverages, studies often compare commercial sports drinks containing mostly corn-derived carbohydrates (CHO) or simple CHO solutions with either mono-, di-, polysaccharides or a mix of isolated compounds [1, 11, 17, 19]. Very few studies have reported using unprocessed CHO sources, such as honey [24] or maple sugar. Maple sap (MW) and syrup (MS) are mostly produced in Quebec, Canada, and appeal to sportspeople for some of their unique characteristics. MS is produced by evaporating maple sap until it reaches a CHO concentration of 66–67% [5, 21]. With a high CHO content, the organic content of MW and MS is around 97% sucrose, with only traces of glucose, fructose and polymers. Both also contain minerals (mainly potassium, calcium and magnesium), phenolic compounds and organic acids [5, 21].

Due to its unique taste and composition, the aim of this study was to compare the sensory characteristics and appreciation of MW and MS with other common CHO products during prolonged exercise. In addition, the effect of ingesting maple-based products on perceived exertion during 2 h of steady-state exercise was investigated.

## Methods

### Subjects

Recreationally and competitively active males (see Table 1) between 18 and 45 years old gave their informed written consent to participate in this study, which was approved by the human health research ethics committee of the University of Montreal (certificate #16–088-CERES-P). This study was part of a larger study, comparing fuel selection between conditions, for which recruitment was mostly done using social media through cycling and triathlon groups, and by word of mouth. All subjects had normal plasma glucose concentrations after a 12-h fast (5.18 ± 0.45 mmol/L) as well as 120 min after ingestion of 75 g of glucose in 300 mL of water (5.43 ± 0.92 mmol/L). Over the course of the study, participants were asked to maintain their regular activity level and to notify investigators of any alteration in their habitual exercise. None of the subjects was a smoker, heavy drinker (< 3 drinks per week), under medication, or taking recreational drugs.

**Table 1:** Detailed information about participants sorted by experimental condition

	n	Age (y)	Body mass (kg)	Height (m)	VO_**2**_max (L.min^**− 1**^)	PPO (W)
**P**	15	29.7 ± 4.9	71.4 ± 10.8	1.77 ± 0.08	4.40 ± 0.62	310 ± 45
**G**	15	28.1 ± 6.2	74.9 ± 7.4	1.78 ± 0.08	4.48 ± 0.54	316 ± 35
**CSD**	15	27.9 ± 6.8	74.7 ± 11.9	1.80 ± 0.10	4.48 ± 0.41	307 ± 38
**MW**	16	29.5 ± 8.9	72.0 ± 7.9	1.77 ± 0.06	4.25 ± 0.54	304 ± 52
**MS**	15	32.3 ± 7.5	75.0 ± 12.7	1.80 ± 0.08	4.24 ± 0.57	301 ± 43

*P* Placebo, *G* Glucose, *CSD* Commercial sports drink, *MW* Maple sap, *MS* Maple syrup, *VO*_*2*_*max* Maximal oxygen consumption, *PPO* Peak power output

### Experimental protocol

Fat-free mass was measured using bioimpedancemetry (SBF-521; Tanita, Tokyo, Japan). Maximal oxygen consumption (VO_2_max) and experimental workload on the cycle ergometer (Excalibur Sport, Lode BV, Groningen, the Netherlands) were determined for each subject using open-circuit spirometry (Cardio-O_2_, MedGraphics, Saint Paul, MN, USA) during a preliminary test session. To avoid any effect of circadian variance and energy availability, the experiments were all performed between 8:30 and 11:30 a.m., after a standardized dinner the day before, and breakfast (mixed diet: 92 kJ/kg/d; 55% CHO, 30% fat and 15% protein) ingested between 7:00 and 8:00 a.m. the morning of the trial. In the 48 h preceding the experiment, subjects were asked to refrain from training or exhaustive exercise.

Following simple randomization, the subjects were assigned to one of five beverage conditions during exercise: maple sap (MW), maple syrup (MS), glucose (G), a commercial sports drink (CSD), or a placebo (P; water sweetened with stevia). The CHO content of the CHO solutions (MW, MS, G and the CSD) was standardized to 60 g/L. MW was provided by the Québec Maple Syrup Producers and had been concentrated to 60 g/L of CHO by reverse osmosis. MS and G were diluted in water the same concentration of CHO as in the CSD (60 g/L). Lemon juice (15 mL/L) and stevia (0.48 g/L) were added to water in the P condition to match the acid taste and the sweetness of the CSD. No electrolytes were added in any of the solutions. Stock solutions were all prepared in advance and stored in a freezer. All beverages needed for the day were prepared in the morning of the experimentation by diluting the stock solution. All solutions were served at approximately 4 °C, in opaque bottles. The exercise protocol consisted of 120 min of steady-state exercise (SSE) on a cycle ergometer at ~ 70% of the maximal power output (MAP; ~ 67% VO_2_max). Beverages were ingested 30 min before (572 mL), immediately before the start and during the exercise period, at every 30 min (286 mL; total of 2 L).

### Measurements

Perceived exertion (RPE) was recorded every 30 min by using the Borg scale [6]. Subjects had to fill a sensory assessment questionnaire pre- and post-SSE. As, to our knowledge, there is no published validated questionnaire to assess sensory perceptions, the questionnaire thus custom-made, inspired by what was done in previous studies [1, 17, 19, 20] and divided in two sections. The first assessed sensory characteristics (acid taste, sweet taste and refreshing taste). These were measured using a 20 cm visual analogue scale labelled with markers appropriate for the sensory property being tested (e.g. “Not sweet” and “Very sweet” for the sweet taste) and then converted to a decimal fraction between 0 and 1 [1, 10, 19, 20]. Another section assessed beverage appreciation (sweet taste, aroma, acid taste, and overall appreciation) using a 9-point hedonic scale. Scale categories varied from “Dislike Extremely” to “Like Extremely” and then converted to a score from 1 to 9 [17, 20].

### Statistical analysis

The distributions of palatability, appreciation and ratings of perceived exertion were assessed for normality using the Kolmogorov-Smirnov test and then analysed using a two-way ANOVA for repeated measures (condition x time). Homogeneity of variances was tested for each comparison using Levene’s test and Tukey’s HSD test was used for post hoc comparisons. Significant differences were identified when *p* < 0.05. All data are reported as mean ± standard deviation. Statistics were computed using the R language and Environment for Statistical Computing, version 3.6.

## Results

### Sensory characteristics

#### Sweet taste

**Fig. 1:**
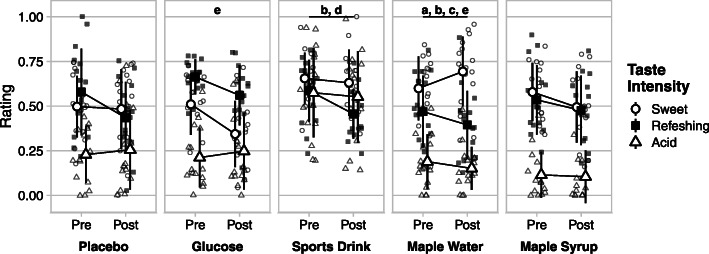
Mean ratings ± SD of sweet, refreshing and acid taste intensity for each experimental condition, pre- and post-exercise (20-cm scale, converted to a fraction from 0 to 1). MW had a higher sweet taste than G (a); MW and CSD both had a higher sweet taste than P (b); MW was less refreshing than G (c); CSD has a higher acid taste than all other conditions (d). Interaction (ingestion x time) showing a significant change in sweet taste (e) over the exercise period. The refreshing taste was significantly higher pre- than post-exercise in all conditions (main effect)

Ratings of sweetness differed among drinks (main effect of ingestion); MW was perceived sweeter than G and P (pre: 0.60 ± 0.19, 0.51 ± 0.17 and 0.50 ± 0.17 and post: 0.69 ± 0.19, 0.34 ± 0.18 and 0.48 ± 0.22; *p* < 0.05, respectively), and CSD was perceived sweeter than G (pre: 0.65 ± 0.14 and 0.51 ± 0.17, post: 0.63 ± 0.19 and 0.34 ± 0.18) (Fig. 1). In addition, an ingestion x time interaction was also found showing ratings decreased for G (0.51 ± 0.17 and 0.34 ± 0.19) and increased for MW (0.60 ± 0.18 and 0.69 ± 0.20) from pre- to post-exercise.

#### Refreshing taste

There was a main effect of ingestion for the refreshing taste, with a higher rating for G than for MW (pre: 0.66 ± 0.09 and 0.47 ± 0.20 and post: 0.56 ± 0.17 and 0.39 ± 0.19; *p* < 0.05) (Fig. 3). A main effect of time was also found for the “refreshing” taste, it was higher before than after exercise (*p* < 0.05).

#### Acid taste

A main effect of ingestion was found in which the CSD was perceived to taste more acid than all other drinks (*p* < 0.05). No time effect was found for the intensity of acid taste.

### Beverage appreciation

#### Overall appreciation

**Fig. 2:**
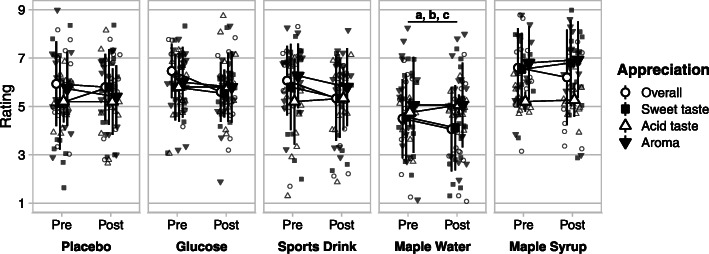
Mean ratings ± SD of overall, sweet taste, acid taste and aroma appreciation for each ingestion condition, pre- and post-exercise (9-point hedonic scale). MW was less appreciated (overall) than all other conditions (a); had a lower appreciation of sweet taste (b) and aroma than MS (c); overall appreciation significantly decreased during exercise (main effect of time) for all ingestions

There was a main effect of ingestion for the overall appreciation, with MW rated lower than all other drinks (*p* < 0.05) (Fig. 2). There was also a main effect of time, with a greater appreciation before than after exercise (*p* < 0.05), regardless on the beverage ingested.

#### Sweet taste, acid taste and aroma

There was a main effect of ingestion for the appreciation of sweet and acid taste, with MS rated higher than MW (pre: 6.5 ± 1.5 vs 4.6 ± 1.8 and post: 6.8 ± 1.8 and 4.1 ± 1.8 for sweet taste and pre: 5.2 ± 0.4 vs 4.8 ± 0.6 and post 5.3 ± 0.7 and 5.1 ± 0.4 for the acid taste of MS and MW, respectively). No effect of time was found for sweet taste, acid taste nor aroma.

### Perception of effort

The ratings of perceived exertion increased over time during SSE for all conditions (main effect of time). There was also a main effect of ingestion for RPE: while no difference was observed between P, G, CSD and MS, subjects ingesting MW reported lower RPE than with P. Although, this result is only significant at 120 min (14.1 ± 2.2 and 16.0 ± 2.0, for MW and P, respectively).

## Discussion

**Fig. 3:**
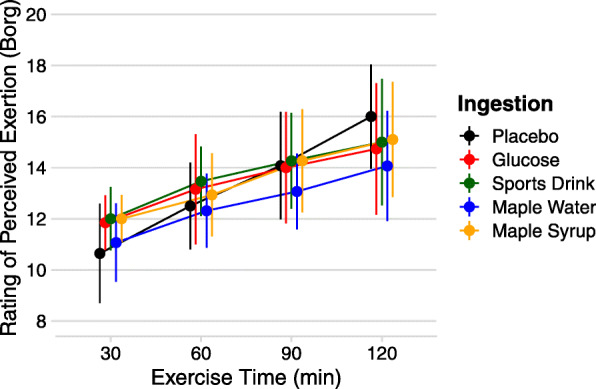
Mean ± SD for RPE every 30 min of the SSE for each experimental condition (Borg scale). RPE is lower with MW ingestion than with P (*)

The aim of the current study was to compare the palatability of maple-based sports drinks to other CHO solutions during prolonged exercise to assess their effect on perceived exertion. The main findings were that MW and CSD conditions were rated significantly sweeter than G and P (Fig. 1). MS ingestion led to a greater overall appreciation compared to MW (Fig. 2). Despite its lower appreciation, subjects receiving MW reported a significantly lower RPE than in other conditions (Fig. 3).

As maple sap and the commercial drink contain mainly sucrose, it could have contributed to the higher sweetness perceived than for the glucose solution or the placebo. Narukawa and colleagues found an increased sensitivity threshold for sucrose during fatigue [18]. However, in the current study, only MW and CSD were rated higher than G and P for sweet taste, although MS also mainly contains sucrose. MS had higher ratings than MW for the appreciation of sweet taste, aroma and overall, having similar ratings for the intensity of the sweet taste (Fig. 2). The reason for these disparities between maple-based sports drinks remains unknown.

In the current study, exercise itself had only minor effects on taste perceptions. Whereas a decrease in overall appreciation after the exercise period was previously reported by Passe et al. [19]. In the latter, ten flavours of CHO drinks (6%) were compared during 180 min of exercise and the overall appreciation of the most acceptable drinks and water decreased; the overall appreciation of the least acceptable drinks increased from resting to exercise and remained stable afterward. Ali et al. [1] have also reported no pre- and post-exercise difference, but an increase in the perceived sweetness for drinks containing various CHO and electrolyte contents, except the placebo, was observed during exercise [1]. Cabanac [7] had suggested that the perceived pleasantness of fluids could be associated with their physiological usefulness and Appleton [2] had indeed reported increased pleasantness with drinks with a lower than higher osmolarity (and CHO content) administered during an hour of exercise at a freely-chosen intensity, varying from moderate to high.

Despite the fact that MW was significantly less appreciated than other beverages, participants who received this beverage reported a significantly lower RPE only at the end of exercise (120 min; Fig. 3). Passe et al. [19] report similar results with no difference in RPE throughout exercise for both the most acceptable and the least acceptable drinks, and many studies corroborate these findings [1, 3, 8, 11, 23]. All found RPE to be increasing with exercise duration, without any effect related to beverage acceptability.

A bitter or sweet taste was previously shown to interact with corticomotor excitability [13] and brain oxygenation [16]. Although, recent results from our laboratory show no difference in brain oxygenation during repeated high-intensity cycling bouts with carbohydrate ingestion (including maple products) or water [12]. In the current study, the lower overall appreciation reported with MW ingestion could be associated with the increased perception of sweet taste during the exercise period. This sweet taste could be less appreciated after a certain time or with increasing effort. Interestingly, MW ingestion led to a slightly lower RPE throughout exercise, although only significant after 120 min. We believe care should be taken when trying to provide mechanistic interpretations for these observations as previous reports on sensory perceptions and their effect on RPE are equivocal.

As suggested by Baker and Jeukendrup [4], non-regulatory factors such as cultural/social preferences could also affect sensory responses and the appreciation of beverages. As such, the current study was performed on recreational and competitive athletes, most of which were originally from or have lived in Quebec for some years and could have been more familiar with the taste of MS than other populations. This could introduce a positive bias towards MS, as Quebecers are strong consumers of maple products [15].

## Conclusions

In summary, both maple sap and a commercial sports drink were perceived to have a sweeter taste than a solution containing only glucose or a placebo. However, sweet taste, aroma and overall appreciation was higher for maple syrup than maple water, and the latter led to a slightly lower perceived exertion after 2 h of exercise. This study was the first to report sensory perceptions associated with the ingestion of maple products during prolonged exercise. More research will be needed to conclude if they provide an advantage over existing products and to assess the potential roles of other bioactive compounds contained in maple products during exercise.

## Data Availability

Please contact the corresponding author for data requests.

## Abbreviations

CHO: Carbohydrates
CSD: Commercial sports drink
MS: Maple syrup
MW: Maple sap
P: Placebo
PPO: Peak power output
RPE: Rating of perceived exertion
SSE: Steady-state exercise
VO_2_max: Maximal oxygen consumption
